# Managing Multiple Medications and Their Packaging for Older People in Home Care Nursing: An Interview Study

**DOI:** 10.3390/healthcare9101265

**Published:** 2021-09-25

**Authors:** Giana Carli Lorenzini

**Affiliations:** Department of Design Sciences, Faculty of Engineering, Lund University, P.O. Box 118, SE 221 00 Lund, Sweden; giana.carli_lorenzini@plog.lth.se

**Keywords:** home care nursing, home health care, interview study, medication management, medication packaging, older people

## Abstract

Home care nurses assist older people in their daily living and well-being, including medication management. Medication management can be challenging for older people with functional constraints and several chronic conditions. This paper presents how home care nurses manage medications and their packaging for older people at their homes. This study followed an explorative qualitative research design, in which semi-structured interviews were conducted with home care nurses in Sweden (n = 14). The study revealed that home care nurses need to coordinate a multitude of interrelated tasks, with documentation being paramount. Regarding medication management, automated systems were preferred, as they reduce medication errors and facilitate dispensing of medications for each patient when compared with analogue systems still in use (i.e., dosing boxes), commonly used by older people. Importantly, the lack of a common journal system for updates on prescribed medication among health care providers and analogue communication still in place creates space for outdated prescription of medications for patients. There are opportunities for further investigation on how technology can help home care nurses in coordinating medication management tasks with other health care providers, and on receiving updates about medication intake by older patients when the nurse is not at their homes.

## 1. Introduction

The aging population, as a global phenomenon, is a positive outcome of advancements in medicine and improved life habits. However, older people are also commonly affected by chronic conditions and geriatric syndromes [1], which lead to daily intake of multiple medications [2]. Whereas some older people might be able to self-care, frail older people living at home with multiple conditions, polypharmacy, and cognitive impairment are at a high risk of adverse outcomes and inappropriate medication intake if not offered appropriate health care services [3,4]. Many developed countries such as Sweden have a large population of older people and an increasing demand for home care services [5]. The Swedish health care system is tax financed, with an eldercare system that follows the guiding principle of aging in place, home care being emphasized [6,7]. In 2019, the municipal cost of elderly care in Sweden reached SEK 132.9 billion [5]. According to the Swedish National Board of Health and Welfare, 379,000 people needed municipal care, including home health care. The majority, 310,000 people, were 65 years or older, most of them women [8].

Home care nurses are therefore a source of support to those older people in managing the necessary care for their daily living [9]. Yet, different than in a hospital setting—where patients often have a passive role in the treatment given to them—at home, these patients have the agency to decide on who is welcome to come in, and to what extent they want to follow the prescribed treatment. Therefore, home care nurses are not only at the patient’s home to perform a list of tasks but also to create the basis for trust and confidence in the treatment, to assess the general well-being of the older person, and to advise on medication intake [10].

Despite its increasing demand and relevance for older people, the scientific landscape of home care is still fragmented [11]. One key aspect of home care nursing that has been under-investigated is the management of multiple medications and their packaging. Medication packaging plays a central role in the protection of the medication from its manufacturing point to the hands of health care staff and patients. Moreover, medication packaging also has other essential functions such as facilitating access to the medication, and providing information about dosage, medication strength, and correct intake [12]. Previous studies showed evidence that packaging can hinder the accessibility of food for older people in hospital settings [13,14]. At the same time, medication packaging has been specifically reported as difficult to use by older people [15,16], with an increased risk of errors at home [17].

By helping several older patients with multiple chronic conditions, home care nurses handle numerous medications, which lifts the interest in knowing how they organize and manage those medications. As care moves to the home of patients [18,19], it is important to investigate the systems in place and possible gaps in the handling of medication for older patients. In addition to that, it is also worth asking what role medication packaging has in helping in the tasks performed and which other essential activities need to be performed for improved health outcomes of older people supported by home care nurses. Studies in this area are scarce and very much needed.

This study investigated the current practice of home care nurses to handle multiple medications and dosing forms for older people that need assistance with their medication intake at home. In particular, this study looked closely at the role of medication packaging, and the systems in place for medication management. Difficulties found with medication packaging and the preferences of the home care nurses were also addressed.

## 2. Materials and Methods

### 2.1. Study Design and Participant Recruitment

This study followed an explorative qualitative research design [20] and was checked following the 32-item list of the Consolidated Criteria for Reporting Qualitative Studies (COREQ) [21]. This study was performed with nurses and district nurses working in home health care in three municipalities in the South of Sweden. We used purposive sampling to approach the home care centers where the nurses worked. A first contact was made with one district nurse informing them about the study via telephone, email, and/or letter. The district nurse then distributed the information internally to the home care nurses, and those interested allowed the researcher to contact them to confirm their willingness to participate and book a suitable time for the interview. The researcher also attended one annual conference for health care practitioners to inform them about the research project and establish contact with district nurses there. Further participants were added through snowballing with the participating home care nurses until data saturation was reached [22]. To be included in the study, the health care worker should have been trained and registered as a nurse and be working in home care nursing for older people at the time of the study for at least six months.

### 2.2. Data Collection

Data were collected through qualitative interviews at the home care centers from January to May 2019. All interviews were conducted individually with each nurse by the same researcher and were audio recorded, complemented by note taking. On the day of the interview, the researcher met the nurse and provided oral and written information about the two-year research project, with a focus on patient-centered medication packaging for older populations. The home care nurse had the opportunity to ask further questions and sign an informed consent form prior to starting the interview. Fourteen home care nurses agreed to participate, and none dropped out of the study.

One interview guide was used with semi-structured questions, organized into four main topics (Table 1), covering general aspects of their profession as well as specific questions about medication management for older patients.

### 2.3. Data Analysis

Transcripts of all interview audio and written notes were analyzed using inductive thematic analysis, which means the coding process is not intended to fit a pre-existing coding frame, and it is therefore data-driven [23].

The interview times ranged from 24 min to 1 h and 25 min. When necessary, participants were contacted again via email or telephone to clarify their answers. Interview transcripts were imported into a qualitative data analysis software (NVivo, QSR International), where they were read entirely several times to permit the researcher to have an overview of all the interview data. Meaningful extracts were then initially coded by one researcher to identify overarching themes and subthemes derived from the data. The thematic analysis was very iterative, with themes and subthemes being refined until reaching consistency within each theme. Examples of how the inductive thematic analysis progressed are presented in Table 2.

Relevant data (i.e., quotations) were selected to illustrate each theme and are presented verbatim in the Results section with a few editions within brackets to facilitate readability.

### 2.4. Ethical Considerations

This study was approved by the Regional Swedish Ethical Board in Lund, Sweden, and it is part of a two-year funded research project focused on the use of multiple medications and patient centricity for older adults. All participants received both oral and written information about the study before they gave their written consent to participate and authorization for audio recording at the beginning of each interview. Each participant received a present card valued SEK 200 at the end of the interview.

### 2.5. Trustworthiness

Trustworthiness was assured using four criteria: credibility, transferability, dependability, and confirmability of study findings [24]. Credibility was promoted through pattern matching of responses and thick descriptions of participants. Transferability and dependability were sustained through the description of participants’ samples and the use of an interview guide, followed by a coding scheme for data analysis. Confirmability was enhanced by recording and transcribing the interviews and having them imported and coded iteratively in a qualitative analysis software.

## 3. Results

In total, fourteen home care nurses were included in this study (female = 11; male = 3), and the mean age was 42 years (range 26–60 years). Their experience as nurses working in home care of older people varied from 6 months to 21 years, indicating a representative and broad sample (Table 3). From the interview data, six main themes were identified: nurse’s role in managing medication for older people, routines in place for medication management, medication management and packaging systems, complaints about medication packaging when managing multiple medications, advice about managing the medication at home, use of technology in medication management.

### 3.1. Nurse’s Role in Managing Medication for Older People

The home care nurses were experienced in managing the medication for older people. As addressed by them in the interviews, it was part of their role to be a key contact point between the older person at home and other health care actors responsible for prescribing the medication, for instance, hospital and primary care staff.

*Nurse 7: I take care of the contact when it comes to health and care. So, I am the responsible person at the primary care level. And we work with health centers and hospitals or clinics, with different things, everything from wound care to medication management and assessments of various kinds. It comes in a lot with medication management and handing over and assessing how the patients cope with their situation.*

However, the home care nurses also commented that their role expanded to other spheres, requiring them to act as “a spider in the web” to promote continuity in the care, to help older people to improve their quality of life with observance of their prescribed treatment, and to avoid deterioration of their health and well-being.

*Nurse 2: I’m like a spider in the web in this nursing network, so to speak. So, I look at their nursing needs to catch if they get worse or if they get better. […] I take care of them, in their care, as a whole thing. And by “whole thing”, I mean their nutrition, pressure ulcer, incontinence protection.*

*Nurse 4: We take actions before maybe a person falls in their home and they get a wound. We try to work one step ahead and we try to see: “Is there any risk in the home right now? Could we avoid this?” […] Medication is to help them and how to get the medications if we’re going to help them take them [the medications]. We help then to plan meetings with the hospital, checkups and we also have a big responsibility to follow up if they go to the doctors, what the doctors choose to do, what treatments we are gonna help them with.*

### 3.2. Routines in Place for Medication Management

In the interviews, the home care nurses were asked to describe their routines for medication management and daily work. From the data, it was possible to apprehend that assistance to each patient is individualized but planned systematically. Routines described by the participating nurses were very similar, starting early in the morning by reviewing what was done last, and going through a list of tasks for the day. The next step then was to delegate those tasks among the team of nurses and assistant nurses, for instance, arranging visits to patients to administer medication, and establishing contact with the primary health care center for updates on the medications.

*Nurse 1: We organize the care, for example, we usually have between 80–100 patients in the area. Maybe many of them need help to get their medications daily, but we are 3 nurses, so we often delegate to assistant nurses and make sure they move on. We instruct [assistant nurses], we have a lot of telephone contact primary health clinics and the hospital. Talks with relatives [of patients]. It’s varied. We do a lot of social work as well. We provide input about illness and how it affects their lives [patients’ lives].*

Documenting tasks performed was considered as a vital part of the work of home care nurses as it permitted continuity in the care. Documentation was conducted mainly as desktop workin a journal system at the home care center, after visits with the patients. The documentation facilitated the follow-up of important changes made in the patient’s treatment and permitted continuity in medication management.

*Nurse 5: The important thing with the documentation is to be able to follow, for example, if it goes forward or if it stands still or if something else is needed, [if] you need to get in touch with the doctor. So that you can follow the development in the documentation*.

### 3.3. Medication Management and Packaging Systems

In general, the home care nurses commented that the patients who receive home care are frail and need help to pick up and manage the medication to be taken, and therefore they authorize the home care nurses to act on their behalf in that matter. Older patients supported by home care nurses often have several medications to take daily, which increases the complexity of their treatment. Due to that, those patients are frequently offered two alternatives to facilitate medication management: dosing boxes or dosing rolls.

#### 3.3.1. Dosing Boxes

Dosing boxes (also known by the branded name *Dosett*) are usually made of hard plastic, with columns for each of day of the week, and rows with times of the day, where tablets are dispensed. The dosing box is organized by the nurse at the home of the patient, where all the medication is gathered and checked upon a list provided by primary health care. Later, the patient becomes in charge of taking the medication from the *Dosett* as prescribed. If the patient cannot or does not want to take the medication from the *Dosett*, an assistant nurse is assigned to help the patient with the medication intake.

*Nurse 2: They buy the Dosett by themselves. Then we go there [at the patient’s home] and dispense [the medications]. They also buy their medications by themselves. If they cannot, then we help them.*

#### 3.3.2. Dosing Rolls

Dosing rolls (also known by the branded name *Apodos*) are part of an automated medication-dispensing system offered by pharmacies, which consists of rolls of medication sachets, where each sachet contains the medication for the day or part of the day [25]. Home care nurses receive the rolls for each patient every second week and perform a sample check to assure the medication is dispensed correctly for each patient. The roll is brought to the patient’s home usually by assistant nurses.

*Nurse 4: The prepackage bags come to us once every two weeks. We get them delivered up here to our office room and then we have a look at them and make sure everything is correct and then we give it to the staff [assistant nurses] which are going home to the patients as well.*

The home care nurses described preferring the *Apodos* system, as it saves time in dispensing the medication, as well as reducing errors. The *Apodos* is also preferred because it permits home care nurses to delegate the distribution of the medication to assistant nurses, who can bring the rolls to patients:

*Nurse 3: When you talk about Apodos, it is a safer system, […] you should work with quality and safety, safe health care.*

*Nurse 7: It has less risk that it [the medication dispensing] would be wrong. And it is easier to adjust [the medication].*

As pointed out, some patients might have their individual preferences for the analogue system of using dosing boxes as they are more accustomed to that. Home care nurses then need to follow the wishes of the patient and help them with the chosen system, even though it is perceived to have an increased risk for medication error in the dispensing of the medication and demands more time for the nurse dispensing the medication.

*Nurse 14: I think [medication] packages are so difficult for them that, in most cases, we divide everything for them [the patients] in doses to make it easy. Some people think Apodos is great, and some people [patients] think they cannot handle Apodos at all. They [the patients] may have incipient dementia and might been very used to their Dosett, and then maybe they want to continue with their Dosett.*

*Nurse 12: Not everyone needs help. We maybe organize the Dosett, but then they [the patients] are happy with it and so they can take [the medications] from the Dosett by themselves. And we take care of the sachets [from Apodos], and then they take their medications by themselves from the sachets. But most [patients] need help to take [the medications] from the Dosett and the sachets.*

In some cases, the medication cannot be dispensed in the Apodos, where it should then be placed in a Dosett or remain in its original packaging, as reported by one nurse:

*Nurse 7: We always check which medications have changed and so on. There are always medications that cannot be dispensed in Apodos. Then we need to put them in a Dosett. Or we keep [them] in the original packaging, if the medication is sensitive to light or something that makes it impossible to dispense in the Dosett.*

Another critical point of having medication in dosing rolls regards updates in the patient’s medication list. When asked about how often these changes occurred, answers varied as patients with multiple conditions that are recurrently at the hospital tend to have more changes in their treatment. The same applies to other patients after appointments with primary care physicians. For the dosing rolls, updates could then be reported automatically in a system, which home care nurses also have access to, guaranteeing the next roll for the patient would already be updated. However, as explained, if there are too many changes in short periods of time, then a dosing box shall be used for a while instead:

*Nurse 13: It happens sometimes that you have so many adjustments with medications, you have to put in and put out, and then you have to treat something, and you have to adjust. And sometimes you wait to start this Apodos roll. Then it is better for us to have it in the Dosett and change the Dosett.*

For dosing boxes, updates in the medication list seemed to be a weak point, as they demand extra control from the home care nurses to assure physicians would send them the updated medication list on paper. Regarding that, some home care nurses mentioned having weekly rounds or frequent calls with primary care to discuss particular cases and stay up to date.

### 3.4. Complaints about Medication Packaging When Managing Multiple Medications

The home care nurses considered multiple medications and their packaging often difficult to be handled by older patients that need assistance at home. That is because these patients are already frail, having less strength in their hands to open medication packaging—in particular, bottles with child-resistant features, blisters that are hard to press or peel off, and packages with small pills that are difficult for an older person to see. For the home care nurses themselves, most of these difficulties were manageable, as they have the necessary strength to remove the medication from the packaging and are used to doing so in their work.

*Nurse 4: It’s tricky for them to open the plastic bottles. Sometimes they have like a child lock, and you have to press a button. That’s hard when you have some older people who don’t have much power in their arms. It is tricky to get a small box and you’re just going to try to take one pill out without them falling over the tables. It’s hard to know how many tablets are still in the package because you cannot see through, so a lot of patients have trouble seeing how much [medication] they have left.*

*Nurse 13: We had one patient, completely clear in her mind, who could take care of everything by herself. The only thing she needed help with was to press out the tablets from blisters, because she had rheumatoid arthritis.*

Changes in the packaging or medication might also have an impact on patients’ and nurses’ identification of the medication. Furthermore, packaging that is too similar can lead to errors in dosage:

*Nurse 12: The medications change names. One time they get one name, and then the next time they get another name. That is why we have to take care of the medications. They [the patients] can no longer handle it. And it is a big risk that they suddenly take twice as much of the same medication, because they think it is a new medication.*

*Nurse 1: It is important that medications with different strengths have different colors on packages or shapes that you can distinguish. For example, a morphine tablet, potent medication, 10 mg and 20 mg look the same. The difference in the packaging is that one is blue and the other is green. But tablets have the same size. The size of the pills and the appearance of the packaging could be adjusted, so that you could see the difference. Even more for patients that take it by themselves.*

Other patients also have some early signs of cognitive impairment, which makes it hard for them to be compliant with their dosing regimen:

*Nurse 13: Birth control pills usually have arrows, and Monday, Tuesday, Wednesday, Thursday, Friday... I do not know if there are other medications with that as well. But if you have forgotten what day it is, it can be difficult.*

### 3.5. Advice about Managing the Medication at Home

The home care nurses commented that older people always have the ultimate decision on accepting to receive help or not. However, most of them seem interested in following their medication regimen and are pleased to be helped.

*Nurse 7: There are always exceptions, those who have their own ideas and do a little as they please. You can recommend and you can inform and so on, but some [patients] still do not follow the advice they have received. But, in most cases, I would say they follow the advice they receive.*

*Nurse 1: Everything is voluntary, you have to explain. They [the patients] must understand their treatment.*

Patients often have questions about new medication or changes to their dosing regimen, which the home care nurses can clarify. However, the home care nurses and assistant nurses also carry out observations of dosing boxes and ask related questions to check they are taking the medication and following the instructions given:

*Nurse 5: Usually, when I am going to instruct patients, I always make a home visit. If it is the case that they will take care of the medications by themselves, then I think it is easier to understand when you sit in front and talk and show it in practice. And then I follow up through a call. Or I ask the assistant nurses to keep an extra eye to see if there are tablets left for example.*

One important aspect highlighted in the interviews is that older patients assisted at home need to have their privacy and own space respected, as exemplified in these quotes:

*Nurse 2: Most older people are generally happy to talk when you are with them, so we talk a little about the weather, and I tell them what I will do at home with them. Because when we go home to them, it’s their home. You should not just go there and say “I’m coming”. You must always ask permission. “Is it okay to come?” And even when you are there, you must clearly state what you are going to do […] because it is their private sphere, their private area. So, it’s not the same as the hospital because in their homes it is them who are active. We [home care nurses] are passive.*

*Nurse 6: […] We must not treat our patients as children, because they are adults, they have their own voice. And you must never forget that. I would not be happy if someone came to my house and told me exactly what to do.*

### 3.6. Use of Technology in Medication Management

As it was mentioned about updates affecting the management of dosing rolls and dosing boxes, medication updates were considered critical, as this included not only coordinated dialogue among different health care providers but also inputs entered in different systems.

Patients discharged from the hospital and/or assessed at primary care who are assigned to receive home health care need to have a list of prescribed medication, where the home care nurses can confirm that the medications are correct. At primary care, updates on prescriptions need to be handled by the physician first, and then home care nurses need to be informed through telephone calls, letters, or fax. Emails are not considered reliable, and a common system for different health care providers is not in place (unless the patient uses *Apodos*). This creates dependability on the physician remembering to inform the home care nurses, and extra control from the home care nurses to assure all updates are communicated and registered in the patient’s medication list.

*Nurse 7: The primary health care center has a system, and then the private care center has another system, and then maybe you have X [name of the system used for Apodos], and then the patient, or the same patient, has maybe three different medication lists that are not integrated into each other and then it becomes very difficult, which one is valid?*

Additionally, when asked about any digital applications to be used when visiting the patients, answers varied. In small municipalities, home care nurses already have access to mobile phones or tablets where they note the tasks performed. However, larger municipalities are still lagging in the implementation of such a system. The reason given is that smaller municipalities have less personnel and need to rely on technology to supply the demand for home care services. They might also be faster in implementing the systems, whereas larger municipalities demand a coordinated effort that takes longer to be fully implemented. Other than that, no mobile technology was reported to be used to facilitate routines of care or control of adherence in the use of medication.

*Nurse 6: No technology at all, just a brain. Physical contact with the patient, talk to them. […]. It sometimes happens that we photograph wounds. It is probably the most advanced we have.*

## 4. Discussion

The complexities in home care nursing are, in part, a consequence of the aging of the population associated with policies of aging in place, which encourage older people to keep living at their homes [5]. Medication management is one important aspect for which many older people may need support either because dosing regimens are too complex with multiple medications to be taken, or because the packaging and dispensing of medication require functional and cognitive capabilities for which external help becomes necessary.

This article presents a unique study of practices of home care nurses in the management of medications of older people, with consideration of the packaging systems in place. Regarding medication packaging, difficulties reported by the home care nurses that affect older people are well known and documented in the literature: for instance, the difficult-to-open child-resistant packages or the problems in distinguishing similar packaging [26,27]. However, different than when managing their self-care and medication at home, older persons assisted by home care nurses often receive help with the dispensing of medication and avoid handling packaging that is too difficult for them. Interestingly, the home care nurses interviewed reported that some older people with capabilities of taking care of themselves might need help only with their medication because of the way the medication packaging is designed, which increases the demand for home care services and limits the independent living of the older person. This supports the understanding that medication packaging should be designed inclusively, with consideration of a wider range of end-users, from experienced health care professionals to older people self-caring at home. As previously addressed by the literature, the balance between having medication packaging that prevents children from accessing medication at home but that is still senior-friendly is a delicate one [28], with many trade-offs along the way from regulatory bodies to manufacturing constraints [29].

In this study, two systems for dispensing were described by the home care nurses: dosing boxes and dosing rolls. Dosing boxes have been used as an analogue system for dispensing medication for decades, with the advantage of avoiding health care staff and patients having to deal with a high number of original packages daily. For older patients, dosing boxes might be particularly important to help them with visual cues that indicate whether the daily dose has been taken or not, facilitating adherence. Dosing boxes were less preferred by the home care nurses as this packaging system was perceived as more passible of medication errors, and it required more time for dispensing. In addition to that, the information provided by the original packaging was then lost, such as the strength of tablets or other features to differentiate among different medications. As it was found in the interviews, dosing boxes may not be suitable for medications that are sensitive and cannot be dispensed out of their original packaging.

The automated medication-dispensing system was a preferred solution by the home care nurses, as it facilitated the correct dispensing of medications and made it possible to delegate medication handling to other nursing staff. This finding is aligned with findings from former research about the *Apodos* system [30]. However, this study did not look into other aspects leading to medication errors: for instance, unnecessary prescriptions, failure of the medication to have the desired efficacy, or even suboptimal therapeutic choice [3]. Dispensing systems such as this also have potential risks of inappropriate drug use that need further investigation [31]. Ultimately, as stressed by the home care nurses, the decision about which system to use (i.e., dosing boxes or dosing rolls) relies on the older person. Moreover, many older people assisted by home care nurses need or want to take the medications by themselves when the nurse is not there, which means other systems to verify adherence to treatment could be in place [32].

Scholars have found that home care nursing has become more complex and multi-faceted than handling multiple medications and their packaging, with home care nurses involved in the coordination of care among interdependent actors [33]. The findings from this study also highlight that home care nurses have a role that is more than just dispensing medication. As revealed in the interviews, home care nurses act as a “a spider in the web”, coordinating a multitude of tasks and interacting with other health care providers. Within this context, digitalization of the work could facilitate home care nursing [34], yet the identified lack of integration between journal systems by different health care providers and the analogue updates of medication prescriptions seems to have an impact on the quality and continuity of care.

Finally, it is important to acknowledge that this study has its limitations. The sample size was adequate for a qualitative study, and no more participants were included once data saturation was reached. Nevertheless, a larger sample should be considered with the intention of quantitatively measuring, for instance, multiple aspects of medication management by home care nurses. Other interesting aspects from our findings could also be further explored, such as the preferences for dosing systems and the potential role of technology in home care nursing. Additionally, in this study, some groups were mentioned but not represented: for instance, the older people receiving assistance from the home care nurses, their relatives, and the other health care providers within the network of primary care services.

## 5. Conclusions

The findings from this study demonstrate the complexities involved in medication management and the use of different packaging systems handled by home care nurses when assisting older people. The role of home care nurses expands to many other spheres associated with the continuity of care in an intricate network of health care providers and systems that are not yet fully integrated. These findings reveal implications in the practice of home care nursing, suggesting opportunities for implementation of technology to follow up adherence to treatment administered to patients, and better integration of care. Accessibility and development of inclusive medication packaging to support complex medication regimens taken at home, as well as the use of automated systems for dispensing, are among relevant topics for further investigation.

## Figures and Tables

**Table 1:** Main topics of the interview guide.

Topic	Example of Questions
Profile	- Gender - Year of birth - What do you work with? How long have you been working with that? - What is your role in the care of older people?
Home care of older people	- Please describe an ordinary day in your life when caring for older people. - How many older people do you visit every day? - What do you talk about when you visit the patient?
Daily treatment and use of medication	- Please tell me how you receive the medication and packages and how you organize each medication for each patient. - Do all the patients need help to take their medication and manage their treatment? - How do you keep track of the updates on the medication changes for the patient?
Following treatment advice	- Do the patients complain or say anything about the medication packaging they have for their medicines? - How do you support your patients to follow your advice and instructions? Can medication packaging help you with that?

**Table 2:** Examples illustrating the inductive thematic analysis.

Meaningful Extracts from the Data	Condensed Meaningful Extract	Initial Themes	Final Theme and Subthemes
“So, for us, it is better if the patient has the *Apodos*, for example. Then it is packed centrally and then it is a bit more like systematic, so to speak. Of course, we can go home and prepare a *Dosett*. But if it’s already packed, like, it also becomes more practical. Then the assistant nurse… they are home and yes, just open the bag and give it.” (Nurse 2)	- Better if the patient has *Apodos* instead of *Dosett* - *Dosett* can still be used and handled by the nurse at the patient’s home - *Apodos* is packed centrally and systematically, and it is practical - Assistant nurse opens the *Apodos* bag and administers the medication to the patients at their homes	Receiving and dispensing the medication: - *Dosett* - *Apodos*	Medication management and packaging systems: - Dosing boxes - Dosing rolls
“We usually start by dispensing the *Dosett* for two weeks. But if they have a lot of medication, we only dispense them for one week at a time. Because, as I said, this is with medication packaging. It takes an enormous amount of time. For a *Dosett* dispensing, if they have twenty medications to dispense, it can take an hour.” (Nurse 14)	- *Dosett* dispensing for two weeks - *Dosett* dispensing for one week, when there are a lot of medications - Twenty medications can take one hour to dispense in the *Dosett*	Receiving and dispensing the medication: - *Dosett*	
“If they are new, then we start with a *Dosett* and divide up the medication in the *Dosett*. And some already know that there is something called *Apodos*. So, they know, or the relatives know about it and think it is great, and then they want to have the *Apodos*. Yes, and that is not a problem. They get to decide, but the routine in our municipality is that most people should have *Apodos*, because it is a much safer medication handling.” (Nurse 13)	- New patients with *Dosett* or *Apodos* - Routine in the municipality to have all new patients with *Apodos*, but patients can decide whether they want to have *Dosett* or *Apodos* - *Apodos* is much safer in medication handling	Receiving and dispensing the medication: - *Dosett* - *Apodos*	

**Table 3:** Profile of participants.

Participant	Gender	Year of Birth	Profession and Work Experience	Work Experience in Home Care Nursing	Home Visits per Day	Number of Older People Under Responsibility of the Nurse	Responsibility for the Same Older People
Nurse 1	Male	1982	Nurse, 10 y	2.5 y	0 to 8	30	Yes
Nurse 2	Female	1964	District nurse, specialized in diabetes, 4 y	2.5 y	2 to 3	20 to 30	Yes
Nurse 3	Female	1962	District nurse, specialized in diabetes, 15 y	6 y	0 to 6	28–30	Yes
Nurse 4	Female	1993	Nurse, 6 mo	6 mo	1 to 2	23	Yes
Nurse 5	Female	1986	Nurse, 4 y	4 y	1	14	Yes
Nurse 6	Male	1959	Nurse, 19 y	5 y	0 to 10	Responsible for the patients when they are released from hospital to home	Yes
Nurse 7	Male	1985	Nurse, 5 y	2.5 y	5 to 10	30	Most often
Nurse 8	Female	1975	Nurse, 15 y, specialized as district nurse, 2 y	9 y	10	150	No, because she comes in when someone is out of work
Nurse 9	Female	1992	Nurse, 5 y	2 y	3 to 15	30	No, takes care when necessary
Nurse 10	Female	1965	Nurse, 34 y	31 y	5 to 10	45	Yes
Nurse 11	Female	1983	Nurse, 13 y	7 y	7	37	Yes
Nurse 12	Female	1970	Nurse, 27 y	21 y	3 to 15	25 to 30	Yes
Nurse 13	Female	1978	Nurse, 5 y	3 y	0 to 3	Responsible for the patients when they are released from hospital to home	Yes
Nurse 14	Female	1982	Nurse, 13 y	7 y	5	30	No

## Data Availability

The data that support the findings of this study are not publicly available due to privacy or ethical restrictions. The full interview guide and COREQ checklist can be provided upon request.
